# Association of *IL17RC* and *COL6A1* genetic polymorphisms with susceptibility to ossification of the thoracic posterior longitudinal ligament in Chinese patients

**DOI:** 10.1186/s13018-018-0817-y

**Published:** 2018-05-15

**Authors:** Peng Wang, Xiao Liu, Bin Zhu, Yunlong Ma, Lei Yong, Ze Teng, Chen Liang, Guanping He, Xiaoguang Liu

**Affiliations:** 1 Department of Orthopedics, Peking University Third Hospital, 49 North Garden Street, Haidian District, Beijing, 100191 People’s Republic of China; 2 The Center for Pain Medicine, Peking University Third Hospital, Beijing, People’s Republic of China; 3 Department of Radiology, Peking University Third Hospital, Beijing, 100191 People’s Republic of China

**Keywords:** Thoracic, Ossification of the posterior longitudinal ligament, IL17RC, COL6A1, Association study

## Abstract

**Background:**

In our previous whole-genome sequencing study of 30 unrelated northern Chinese Han patients, we identified six single nucleotide polymorphisms (SNPs) in the interleukin 17 receptor C (IL17RC) and collagen type VI α1 chain (COL6A1) genes that were potentially associated with thoracic ossification of the posterior longitudinal ligament (T-OPLL). To determine whether these six SNPs are associated with susceptibility to T-OPLL in the northern Chinese Han population, we performed a case-control association study to confirm specific susceptible loci in the expanded samples.

**Methods:**

The six SNPs in the *IL17RC* and *COL6A1* genes were analyzed in 200 northern Chinese individuals (100 patients and 100 control subjects) using the Sequenom system.

**Results:**

The genotype distributions and allele frequencies of each SNP in the control and patient groups were compared. rs201153092, rs13051496, rs199772854, rs76999397, and rs189013166 showed potential pathogenic loci for T-OPLL in the northern Chinese Han population, whereas rs151158105 did not. At the genotype level, the differences in the genotype frequencies of rs201153092, rs13051496, rs199772854, rs76999397, and rs189013166 between T-OPLL cases and controls reached statistical significance.

**Conclusions:**

To the best of our knowledge, this is the first association study of susceptibility genes in Han Chinese patients with T-OPLL. The results revealed five SNPs in the *IL17RC* and *COL6A1* genes that represented potentially pathogenic mutations in patients with T-OPLL.

## Background

Ossification of the posterior longitudinal ligament (OPLL), which involves pathological heterotopic ossification of this region, can lead to symptoms of spinal cord compression and radiculopathy in affected patients. The disease has a marked ethnic predilection, with epidemiological studies suggesting that OPLL is found almost exclusively in Japanese, Chinese, and Korean individuals [1]. Approximately 70% of OPLL cases affect the cervical spine, whereas 15% occur in the thoracic spine [2]. Ohtsuka et al. reported that the prevalence of thoracic OPLL (T-OPLL) in Japanese individuals is 0.8%, which is lower than that of cervical OPLL (3.2%) [3]. Although the most common site of OPLL is the cervical spine, T-OPLL is considered more severe.

As T-OPLL is slowly progressive, the majority of patients develop symptoms only when the disease progresses to an advanced stage, when severe spinal cord injury occurs. Clinical manifestations include sensory disturbances, dyskinesia, fecal and urinary incontinence, and even paraplegia. The disability rate in T-OPLL is much higher than that in cervical OPLL. Currently, treatment approaches for T-OPLL are limited, and surgery is the only effective treatment. However, owing to the unusual blood supply and anatomical structure associated with this disease, surgery for T-OPLL is complicated; it is difficult to avoid surgical complications, and the risk of paralysis is extremely high. The optimal surgical methods for T-OPLL, as well as the timing of the surgery, remain controversial, thus it remains a challenge for clinicians to perform timely and effective treatment of the disease [4].

Genetics are considered to be a predominant factor in the etiology of OPLL. Several East Asian groups have performed candidate gene linkage and association studies, which have suggested that a number of polymorphisms in osteogenic genes are associated with the occurrence and development of cervical OPLL. To date, 16 genes/loci that are linked to OPLL susceptibility have been reported [5], including toll-like receptor 5 (*TLR5*) [6], collagen type XII α2 chain (*COL11A2*) [7], *COL6A1* [8–10], runt-related transcription factor 2 (*RUNX2*) [11], bone morphogenetic protein 2 (*BMP2*) [12], bone morphogenetic protein 4 (*BMP4*) [13], transforming growth factor β1 (*TGFB1*) [14], interleukin 1β (*IL1B*) [15], and interleukin 15 receptor subunit α (*IL15RA*) [16]. However, these findings have not been sufficiently reproducible, and usually only one SNP was examined to evaluate the gene, and thus there is a lack of linkage disequilibrium (LD) studies on multiple single nucleotide polymorphisms (SNPs) [17]. In addition, no genetic studies have assessed the causes of T-OPLL.

In our previous whole-genome sequencing (WGS) study of 30 unrelated northern Chinese Han patients, four different algorithms [including SIFT(http://sift.jcvi.org/) [18], PolyPhen-2 (http://genetics.bwh.harvard.edu/pph2/) [19], MutationTaster (http://www.mutationtaster.org/) [20], and GERP++ (http://mendel.stanford.edu/SidowLab/downloads/gerp/) [21]] were used to predict deleterious variations at different SNP loci. We found that rs201153092 was predicted to be deleterious by four algorithms, rs199772854 was predicted by three algorithms, and rs151158105, rs13051496, rs76999397, and rs189013166 were predicted by two algorithms (Table 1). In the present study, we aimed to identify genetic factors for T-OPLL by performing a case-control study of six SNPs in two genes (*COL6A1* and *IL17RC*) potentially associated with susceptibility to T-OPLL in the northern Chinese Han population.

**Table 1:** Mutation findings for 30 patients with T-OPLL by WGS

Gene	SNP ID	Chromosome	Nucleotide change	Protein change	1000G (EAS)	SIFT	PP2	MutationTaster	GERR++
COL6A1	rs201153092	21	c.1534G>A	p.Gly512Ser	0	D	D	D	R
COL6A1	rs13051496	21	c.2669C>T	p.Ser890Leu	0	T	B	D	R
COL6A1	rs151158105	21	c.1298G>A	p.Arg433Gln	0	D	B	N	R
IL17RC	rs199772854	3	c.2275C>A	p.Leu759Ile	0	D	D	N	R
IL17RC	rs76999397	3	c.1908G>A	p.Ala636Ala	0.00378	D	B	N	R
IL17RC	rs189013166	3	c.2238G>A	p.Gly746Gly	0	D	B	N	R

1000G (EAS), 1000 Genomes (Asian); SIFT (*D* deleterious, *T* tolerated); PP2, Polyphen-2 (*D* probably damaging, *P* possibly damaging, *B* benign); MutationTaster (*D* disease causing, *N* polymorphism); GERP++ (*R* rejected substitutions, *S* substitutions)

## Methods

### Case selection and grouping

The study protocol was approved by the Ethics Committee for Human Subjects of the Peking University Third Hospital. Informed consent was provided by all participating individuals. A total of 100 unrelated northern Chinese Han T-OPLL patients with myelopathy and/or neurological dysfunction [47 men (mean age, 51.00 ± 3.30 years) and 53 women (mean age, 52.34 ± 6.09 years)] and 100 sex-matched unrelated healthy controls [45 men (mean age, 55.84 ± 3.06 years) and 55 women (mean age, 54.36 ± 5.69 years)] were enrolled in this study between January 2010 and October 2016. Diagnosis of T-OPLL was performed by specialists based on clinical symptoms and radiological examinations (including computed tomography and magnetic resonance imaging) of the thoracic spine. The appearance of OPLL observed on the radiographs was classified into four subtypes: (i) segmental, (ii) continuous, (iii) mixed, and (iv) local [22]. Individuals who had lumbar spondylolisthesis, ankylosing spondylitis, diffuse idiopathic skeletal hyperostosis, and disc herniation of the thoracic spine were included in the study, and none were taking any drugs known to affect bone or calcium metabolism.

### SNP selection and genotyping

EDTA-anticoagulated peripheral blood samples were obtained from all participants for DNA extraction. Genomic DNA samples were extracted from peripheral leukocytes with the standard procedure using a Wizard Genomic DNA Purification Kit (Promega Corporation, Madison, WI, USA). Six SNPs distributed among the two genes were selected based on findings from our previous WGS studies. The polymerase chain reaction (PCR) fragments were submitted for Sanger sequencing at the Beijing Genomics Institute, and the forward and reverse sequence reads were assembled and analyzed in DNAStar version 7.1. Details of the six studied SNPs and the primer sequences are listed in Table 2. The six primers were used for PCR as described previously [23]. PCR was performed with 20 ng genomic DNA per 15-μl reaction mixture, containing 0.2 μM of each primer, 200 μM of deoxyribonucleotides, 50 mM KCl, 10 mM Tris HCl (pH 8.3), 1.5 mM MgCl_2_, and 0.5 units of Taq DNA polymerase in a DNA Gradient PCR machine (Bio-Rad Laboratories, Inc., Hercules, CA, USA). The thermocycling conditions were as follows: initial denaturation at 95 °C for 10 min; followed by 35 cycles of 95 °C for 30 s, annealing at an assay-specific temperature (48 °C to 65 °C) for 45 s, and elongation at 72 °C for 45 s; and a final terminal elongation step at 72 °C for 5 min. The PCR products were analyzed by direct sequencing using a BigDye Terminator v3.1 Cycle Sequencing Kit (Thermo Fisher Scientific, Inc., Waltham, MA, USA) with POP-7™ Polymer in a 3730XL DNA Analyzer with Sequencing Analysis Software version 5.2 (Thermo Fisher Scientific, Inc., Waltham, MA, USA).

**Table 2:** Details of the six SNPs in *COL6A1* and *IL17RC* and their associated primers

Gene	SNP ID	Nucleotide substitution (M/m)	Primer sequence
*COL6A1*	rs201153092	G/A	Forward 5′-TGAAAGGGTGAGTGTCCAA-3′ Reverse 5′-GTGCCCAGTCCACTAAAGAG-3′
*COL6A1*	rs13051496	C/T	Forward 5′-AGCCACAACTTTGACACCA-3′ Reverse 5′-GAAGCGGGTCACATAGCC-3′
*COL6A1*	rs151158105	G/A	Forward 5′-CCTCCTGCCCAAGACA-3′ Reverse 5′-TCCAAAGAAGAACCCAAGC-3′
*IL17RC*	rs199772854	C/A	Forward 5′-CCCAACTGCCAGACTTCCT-3′ Reverse 5′-GCCACAGCCTGCGTAAAA-3′
*IL17RC*	rs76999397	G/A	Forward 5′-GGCTCTGCTCCTCTACTCAG-3′ Reverse 5′-AATGACGTTTGCCAGCCC-3′
*IL17RC*	rs189013166	G/A	Forward 5′-GGCTCTGCTCCTCTACTCAG-3′ Reverse 5′-AATGACGTTTGCCAGCCC-3′

### Statistical analysis

All statistical analyses were performed using SPSS v17.0 software (SPSS, Inc., Chicago, IL, USA). The *χ*^2^ test was used to determine whether individual variants were in equilibrium at each locus in the population (Hardy-Weinberg equilibrium), as well as the genotypic and allelic distributions. Pairwise LD was calculated as D′ and *R*^2^ using Haploview version 4.2. The Pearson’s *χ*^2^ test was used to determine the correlation between the variants and the disease based on the odds ratios (ORs) with 95% confidence intervals (95% CI). All statistical tests were two-sided, and significance was set at *P* < 0.05.

## Results

Six SNPs were genotyped among northern Chinese Han subjects. With the exception of rs151158105, which was incompatible with the Hardy-Weinberg equilibrium (*P* = 0.005), the remaining five SNPs were in Hardy-Weinberg equilibrium (*P >* 0.05).

The allelic frequencies and genotype distributions of the six studied SNPs are shown in Tables 3 and 4. rs201153092, rs13051496, rs199772854, rs76999397, and rs189013166 showed significant associations with T-OPLL (*P <* 0.05). The A allele frequency of rs201153092 was higher in T-OPLL cases (8.5%) than in controls (0.5%). The T allele frequency of rs13051496 was higher in T-OPLL cases (5.5%) than in controls (1.0%). The A allele frequency of rs199772854 was higher in T-OPLL cases (6%) than in controls (1.0%). The A allele frequency of rs76999397 was higher in T-OPLL cases (4.5%) than in controls (1.0%). The A allele frequency of rs189013166 was higher in T-OPLL cases (4.0%) than in controls (0.5%).

**Table 3:** Allelic frequencies of the six SNPs

SNP ID	*N*	Allele frequency (%)		Versus control	
		Major allele	Minor allele	*P* value^a^	Odds ratio (95% CI)
rs201153092		G	A		
Cases	100	91.50	8.50	0.000114	18.49 (2.436–140.3)
Controls	100	99.50	0.50		
rs13051496		C	T		
Cases	100	94.50	5.50	0.01116	5.762 (1.26–26.34)
Controls	100	99.00	1.00		
rs151158105		G	A		
Cases	100	99.50	0.50	0.3167	–
Controls	100	100.00	0.00		
rs199772854		C	A		
Cases	100	94.00	6.00	0.006515	6.319 (1.396–28.61)
Controls	100	99.00	1.00		
rs76999397		G	A		
Cases	100	95.50	4.50	0.03234	4.665 (0.9951–21.87)
Controls	100	99.00	1.00		
rs189013166		G	A		
Cases	100	96.00	4.00	0.01827	8.292 (1.027–66.92)
Controls	100	99.50	0.50		

*N* number of subjects

^a^Allele frequency difference between cases and controls

**Table 4:** Genotype frequencies of the six SNPs

SNP ID	*N*	Genotype frequency (%)			Versus control	
		MM	Mm	mm	*P* value^a^	*P* value^b^
rs201153092		GG	GA	AA		
Cases	100	83	17	0	–	0.00007707
Controls	100	99	1	0		
rs13051496		CC	CT	TT		
Cases	100	89	11	0	–	0.009838
Controls	100	98	2	0		
rs151158105		GG	GA	AA		
Cases	100	99	1	0	–	0.3161
Controls	100	100	0	0		
rs199772854		CC	CA	AA		
Cases	100	88	12	0	–	0.005582
Controls	100	98	2	0		
rs76999397		GG	GA	AA		
Cases	100	90.91	9.09	0	–	0.02992
Controls	100	98	2	0		
rs189013166		GG	GA	AA		
Cases	100	92	8	0	–	0.01696
Controls	100	99	1	0		

*N* number of subjects

^a^Dominant model: (major allele homozygote + heterozygote)/minor allele homozygote

^b^Recessive model: major allele homozygote/(minor allele homozygote + heterozygote)

At the genotype level, the differences in the genotype frequencies at rs201153092, rs13051496, rs199772854, rs76999397, and rs189013166 reached statistical significance between T-OPLL cases and controls (recessive model; *P* = 0.00007707, *P* = 0.009838, *P* = 0.005582, *P* = 0.02992, and *P* = 0.01696, respectively). No significant difference was observed in the genotype or allele frequencies of rs151158105 between T-OPLL cases and controls. The LD coefficients among rs201153092, rs151158105, and rs13051496 ranged from 0.91 to 1, and the *R*^2^ value ranged from 0.05 to 0.6 (Table 5). The LD coefficients among rs199772854, rs76999397, and rs189013166 were 1, and the *R*^2^ value ranged from 0.63 to 0.81 (Table 6).

**Table 5:** Linkage disequilibrium statistics among *IL17RC* SNPs (D′/*R*^2^)

SNP ID	rs76999397	rs189013166	rs199772854
rs76999397	–	0.81	0.78
rs189013166	1	–	0.63
rs199772854	1	1	–

**Table 6:** Linkage disequilibrium statistics among *COL6A1* SNPs (D′/*R*^2^)

SNP ID	rs151158105	rs201153092	rs13051496
rs151158105	–	0.05	0.07
rs201153092	1	–	0.6
rs13051496	1	0.91	–

## Discussion

OPLL is a condition that is characterized by the calcification of the soft tissues that connect the bones of the spine. Although the exact underlying cause is currently unknown, it has been suggested that it is a multifactorial condition that is influenced by several different genetic and environmental factors [17]. The accumulation of deleterious missense mutations in individual human genomes has been proposed to form the genetic basis of complex diseases [24], and WGS is considered to be a promising approach for the study of such diseases [5]. In the present study, we performed association studies to confirm specific susceptibility loci in expanded samples.

In this study, rs201153092 and rs13051496 in *COL6A1* were potential pathogenic loci for T-OPLL in the northern Chinese Han population. According to our data, the A allele frequency of rs201153092 was higher in T-OPLL cases than controls, which implied that the A allele could be a risk factor for the genetic susceptibility to T-OPLL and that the “GA” genotype enhanced the probability of the occurrence of T-OPLL. The T allele frequency of rs13051496 was higher in T-OPLL cases than in controls, which implied that the T allele could be a risk factor for the genetic susceptibility to T-OPLL and that the “CT” genotype enhanced the probability of T-OPLL susceptibility.

*COL6A1*, which encodes the α1 chain of type VI collagen, is located on chromosome 21q22.3 and spans approximately 23.3 kbp. *COL6A1* encodes an extracellular matrix protein that may serve as a scaffold for osteoblastic or preosteoblastic cells or chondrocytes that subsequently undergo membranous or endochondral ossification [25]. Although the functional impacts of rs201153092 and rs13051496 in *COL6A1* in bone metabolism remain elusive, the molecular variants of these extracellular proteins may be implicated in the ectopic bone formation observed in T-OPLL patients [26].

rs199772854, rs76999397, and rs189013166 in *IL17RC* were potential pathogenic loci for T-OPLL in the northern Chinese Han population. The current study showed that the A allele frequency of rs199772854 was higher in T-OPLL cases than in controls, which implied that the A allele could be a risk factor for the genetic susceptibility to T-OPLL and that the “CA” genotype enhanced the probability of T-OPLL susceptibility. The A allele frequency of rs76999397 was higher in T-OPLL cases than in controls, which implied that the A allele could be a risk factor for the genetic susceptibility to T-OPLL and that the ‘GA’ genotype was enhance the probability of T-OPLL susceptibility. In addition, the A allele frequency of rs189013166 was higher in T-OPLL cases than controls, which implied that the A allele could be a risk factor for the genetic susceptibility to T-OPLL and that the ‘GA’ genotype was enhance the probability of T-OPLL susceptibility.

The *IL17RC* gene encodes a single-pass type I transmembrane protein located between the chromosomal regions 3p25.3 and 3p24.1, spanning approximately 16.55 kbp [27]. *IL17RC* has been implicated as an important regulator of bone metabolism that accelerates osteoblast differentiation. A recent study indicated that the IL17RC protein plays an indispensable role in osteoblastogenesis [28]. Moreover, the TGF-β signaling pathway is known to be associated with the initiation of ectopic ossification [29]. The *IL17RC* gene may also be involved in bone metabolism through canonical TGF-β signaling. This study provides a basis for the study of the effects of these SNPs on OPLL in the future.

There were some limitations in the present study. First, the power of bioinformatics analyses and damaging-variant prediction algorithms are limited. The results of allelic frequencies and genotype distributions showed that there is no homogeneity of the disease-related allele and homozygous allies of pathological related minor allies in any of the T-OPLL patients, suggesting that the T-OPLL disease has highly genetic heterogeneities and complex pathogenesis. In addition, we lacked studies of co-efficiency between the allies of these two genes. To accomplish these issues, further genetic and functional studies, including studies with more participants of other ethnicities, are needed to confirm these positive findings.

## Conclusions

To the best of our knowledge, this is the first association study of T-OPLL susceptibility genes in Han Chinese patients. From the current analysis, we identified five new potential pathogenic loci for T-OPLL: rs201153092 and rs13051496 in the *COL6A1* gene; and rs199772854, rs76999397, and rs189013166 in the *IL17RC* gene. The results of the current study may be beneficial for clarifying the molecular etiology of T-OPLL.

Further genetic studies with more participants of other ethnicities are required to confirm these positive findings, and functional studies will further our understanding of this obscure disease.

## Abbreviations

BMP2: Bone morphogenetic protein 2
BMP4: Bone morphogenetic protein 4
COL11A2: Collagen type XII α2 chain
COL6A1: Collagen type VI α1 chain
IL15RA: Interleukin 15 receptor subunit α
IL17RC: Interleukin 17 receptor C
IL1B: Interleukin 1β
OPLL: Ossification of the posterior longitudinal ligament;
RUNX2: Runt-related transcription factor 2
SNPs: Single nucleotide polymorphisms
TGFB1: Transforming growth factor β1
TLR5: Toll-like receptor 5
T-OPLL: Thoracic ossification of the posterior longitudinal ligament
